# Common Methods for Phylogenetic Tree Construction and Their Implementation in R

**DOI:** 10.3390/bioengineering11050480

**Published:** 2024-05-11

**Authors:** Yue Zou, Zixuan Zhang, Yujie Zeng, Hanyue Hu, Youjin Hao, Sheng Huang, Bo Li

**Affiliations:** 1 College of Life Sciences, Chongqing Normal University, Chongqing 401331, China; 2021051301110@stu.cqnu.edu.cn (Y.Z.); 2020051305046@stu.cqnu.edu.cn (Z.Z.); zengyj2000118@163.com (Y.Z.); 2023210513008@stu.cqnu.edu.cn (H.H.); haoyoujin@hotmail.com (Y.H.); 2 Animal Nutrition Institute, Chongqing Academy of Animal Science, Chongqing 402460, China

**Keywords:** phylogenetic tree, neighbor-joining method, maximum parsimony method, maximum likelihood method, Bayesian method, tree integration, R language

## Abstract

A phylogenetic tree can reflect the evolutionary relationships between species or gene families, and they play a critical role in modern biological research. In this review, we summarize common methods for constructing phylogenetic trees, including distance methods, maximum parsimony, maximum likelihood, Bayesian inference, and tree-integration methods (supermatrix and supertree). Here we discuss the advantages, shortcomings, and applications of each method and offer relevant codes to construct phylogenetic trees from molecular data using packages and algorithms in R. This review aims to provide comprehensive guidance and reference for researchers seeking to construct phylogenetic trees while also promoting further development and innovation in this field. By offering a clear and concise overview of the different methods available, we hope to enable researchers to select the most appropriate approach for their specific research questions and datasets.

## 1. Introduction

**Figure 1:**
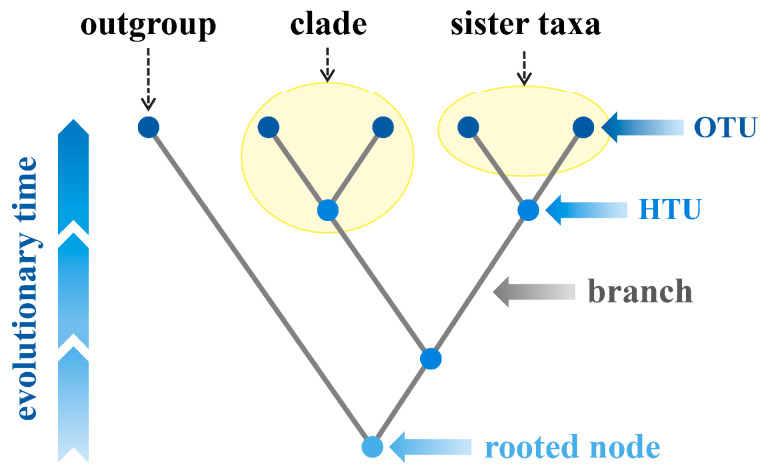
The general structure of a phylogenetic tree. The abbreviations in the figure are as follows: OTU, operational taxonomic unit; HTU, hypothetical taxonomic unit. All figures in this review were drawn by Yue Zou, using Microsoft PowerPoint 2010 and Adobe Illustrator 26.2.1.

A phylogenetic tree, also known as a cladogram, tree of life, or evolutionary tree, is a graphical representation resembling a tree that illustrates the evolutionary and phylogenetic relationships between biological taxa based on their physical or genetic characteristics [1,2,3]. Comprising nodes and branches, a phylogenetic tree uses nodes to stand for taxonomic units and branches to depict estimated time relationships between these units [4,5]. As shown in Figure 1, there exist two types of nodes in a phylogenetic tree: internal nodes and external nodes (leaf nodes). Internal nodes are hypothetical taxonomic units (HTUs), with the topmost internal node called the root node, symbolizing the most recent common ancestor of all leaf nodes, marking the starting point of evolution. External nodes represent operational taxonomic units (OTUs), typically indicating species but also capable of representing extinct lineages or fossil endpoints [6,7]. The evolutionary clade within the phylogenetic tree encompasses a node and all lineages stemming from it. Depending on the different topological structures, phylogenetic trees can be categorized into rooted trees and unrooted trees: rooted trees have a root node from which the rest of the tree diverges, indicating an evolutionary direction. In contrast, unrooted trees lack a root node and only illustrate relationships between nodes without suggesting any evolutionary direction [8].

The phylogenetic tree visually presents the evolutionary history and phylogenetic relationships between different taxonomic units, facilitating people’s understanding of the causes of species’ morphological diversity and evolutionary patterns [9]. On the one hand, a phylogenetic tree can drive the development of phylogenetic systematics [10]. On the other hand, it can help reveal patterns such as genetic structure, gene flow, and genetic drift among populations, providing important clues for population genetics research [11,12]. 

## 2. The Popular Methods for Inferring Phylogenetic Trees

**Figure 2:**
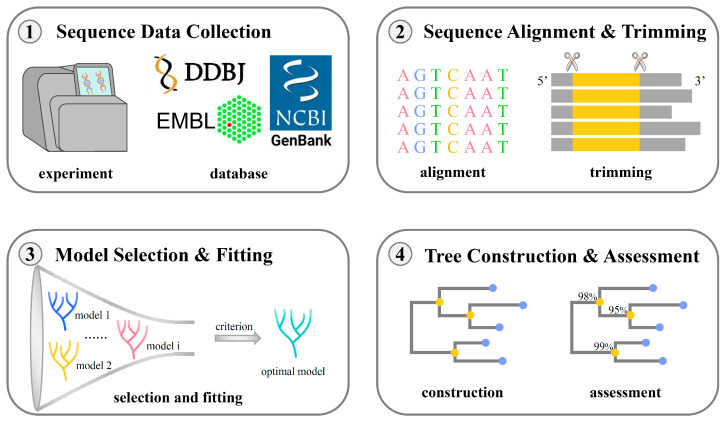
The construction workflow of a phylogenetic tree. A classic process used to build the evolutionary tree usually contains the following steps: (**1**) sequence data collection, (**2**) sequence alignment and trimming, (**3**) model selection and fitting, as well as (**4**) tree construction and evaluation. The abbreviations in the figure are as follows: DDBJ, DNA Data Bank of Japan; EMBL, European Molecular Biology Laboratory; NCBI, National Center for Biotechnology Information.

Before the advent of DNA sequencing technologies, biologists typically relied on traditional taxonomic features such as biological morphology and traits to infer phylogenetic trees (regarded as species trees). However, with the development of sequencing technologies, a large amount of accumulated gene sequences became the basic data for inferring species trees [13,14]. Since genes and species often coevolve, they often exhibit similar evolutionary patterns, allowing gene trees to be used for inferring species trees [15,16]. Figure 2 illustrates the general process of constructing a phylogenetic tree starting from gene sequences, including steps such as sequence collection, sequence alignment, model selection, tree inference, and tree evaluation. Typically, researchers first collect homologous DNA (or protein) sequences through experiments or public databases (such as GenBank, EMBL, DDBJ) and then perform sequence alignment. Accurate alignment results form the basis for inferring evolutionary relationships, and multiple methods are commonly used in practice to generate consistent results [17]. It should be noted that the aligned sequences need to be precisely trimmed before inferring the tree structure to remove unreliable regions that may affect subsequent analysis [18]. Insufficient trimming may introduce noise, while excessive trimming may remove genuine signals that help with phylogenetic analysis [19,20]. Once the sequence alignment is completed, researchers then select appropriate algorithms for phylogenetic tree inference [21].

There are two main categories of methods used for phylogenetic tree inference [22]: (1) Distance-based methods (such as the NJ method and the UPGMA method) [23]. These methods first convert the feature matrix into a distance matrix to represent the evolutionary distances between pairs of species, and then combine clustering algorithms to analyze the species under study and infer the phylogenetic tree [24]. (2) Character-based methods (such as the parsimony method and the likelihood method) [25,26]. These methods typically generate a large number of hypothetical trees based on an algorithm (such as the MP method, ML method, and BI method) and then induce an optimal tree according to certain criteria [27]. Among them, the parsimony method has no explicit model assumptions, while the likelihood method has a specific fixed sequence evolution model and likelihood function [28]. Distance methods always produce a single evolutionary tree, while parsimony and likelihood methods involve numerous hypothetical trees before producing the optimal tree. The characteristics of these common tree-building methods are shown in Table 1.

### 2.1. Distance-Based Method

**Figure 3:**
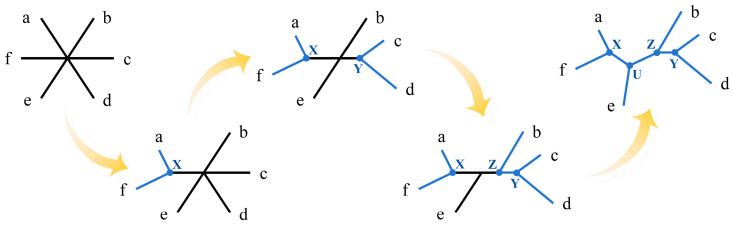
Fundamentals of the neighbor-joining methods for constructing phylogenetic trees. The abbreviations in the figure are as follows: a, b, c, d, e, and f represent different operational taxonomic units, and X, Y, Z, and U represent different hypothetical taxonomic units.

Distance-based methods are the simplest approach for constructing phylogenetic trees. They transform the molecular feature matrix of different species into a distance matrix and then use clustering algorithms to classify these species and infer the evolutionary relationships [29]. Representative methods in this category include neighbor-joining (NJ) and unweighted pair group method with arithmetic mean (UPGMA) [30]. The NJ method, created by Naruya Saitou and Masatoshi Nei in 1987, is an agglomerative clustering algorithm [31]. The tree-building process is illustrated in Figure 3. Firstly, an initial distance matrix is constructed based on similarity measures between sequences. In practice, users can choose appropriate distance metrics (such as the Hamming distance, Jaccard distance, Euclidean distance, and Manhattan distance) according to the characteristics of the sequence data and the research question. Then, an initial tree for an unrooted star-like network is created based on the initial matrix. Subsequently, the distance matrix is updated by merging the two nodes with the smallest distance, and a new node connecting these two clusters is created in the tree topology. This new node is connected to the central node, updating the tree topology. This step is repeated until only one cluster remains, resulting in the NJ tree. 

The NJ method has high accuracy and fewer assumptions when reconstructing phylogenetic trees. It also has a faster computation speed. It uses a stepwise construction approach to build the evolutionary tree instead of searching for the optimal tree [32,33]. As the number of sequences increases, the number of potential topologies to be examined grows exponentially, making the probability of finding the best tree rapidly decrease. At this point, the advantages of the NJ method over the parsimony method and likelihood method become more evident, leading to its wide usage in analyzing large datasets [34]. Additionally, the neighbor-joining method allows for different branch lengths between sequences and permits multiple substitutions. However, converting sequence differences into a distance matrix may result in a reduction of sequence information when the sequence divergence is substantial [35].

### 2.2. Maximum Parsimony (MP) Method

**Figure 4:**
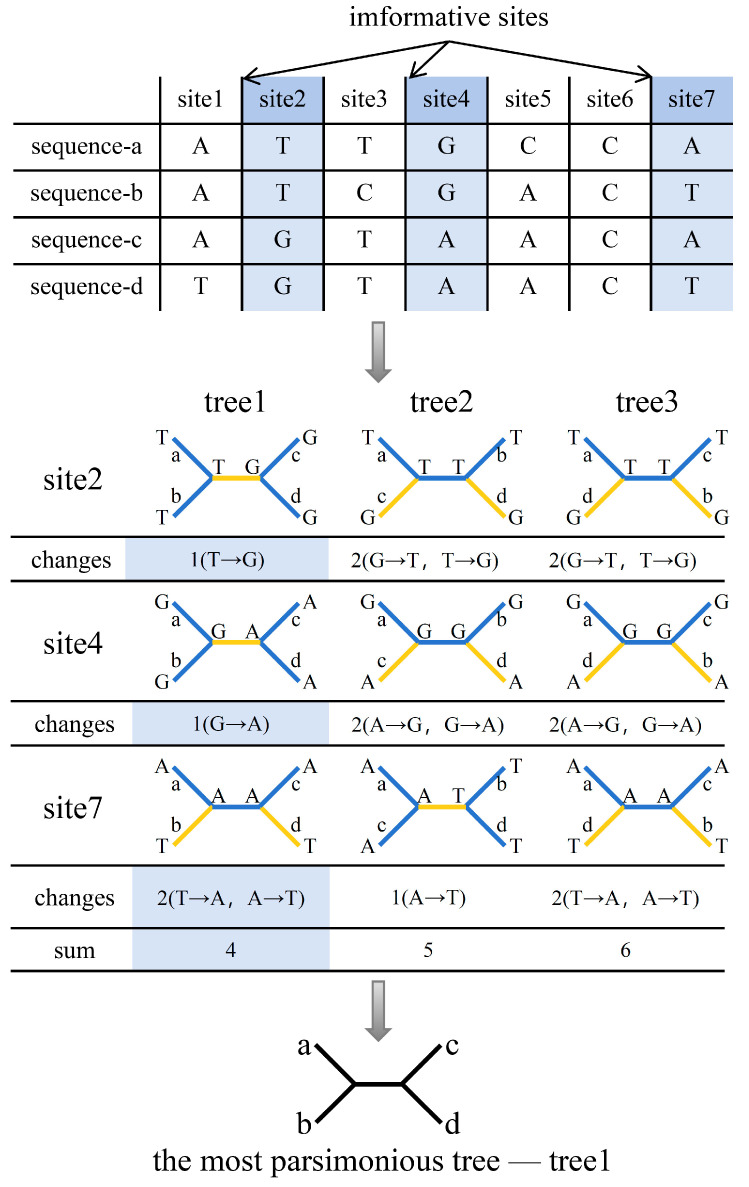
Fundamentals of the maximum parsimony method for constructing phylogenetic trees. The abbreviations in the figure are as follows: a, b, c, and d represent sequences a, b, c, and d, respectively.

Maximum parsimony (MP) is a phylogenetic tree reconstruction algorithm based on the principle of Occam’s razor, aiming to infer the evolutionary tree by minimizing the number of evolutionary steps required to explain the dataset [36]. This method was proposed by James S. Farris and Walter M. Fitch in 1970–1971 [37,38]. MP primarily considers informative sites and requires the identification of informative sites in the sequences before tree construction [39]. Figure 4 illustrates the basic process of constructing an evolutionary tree using MP. Taking DNA sequences as an example, a site is considered informative if it has at least two different nucleotides and each nucleotide appears in at least two of the studied sequences. By using informative sites, all possible tree topologies (constructing the tree space) are searched, and the minimum number of nucleotide substitutions for each topology is counted to obtain the most parsimonious tree. In simple terms, it involves finding the tree that minimizes the total number of substitutions across all informative sites. Increasing the number of taxa during tree construction leads to a rapid increase in the number of possible tree topologies. Therefore, when there are fewer taxa, exhaustive search algorithms are often used, while branch-and-bound and heuristic search algorithms are used to improve computational efficiency when there are more taxa [35]. Popular heuristic algorithms include Subtree Pruning and Regrafting (SPR) and Nearest Neighbor Interchange (NNI) [40]. MP may result in multiple equally parsimonious trees, so it is common practice to construct a consensus tree to represent the final result. This is achieved by treating consistent branch points in all trees as binary branches, converting partially consistent branch points into internal nodes connecting multiple branches, or selecting the most frequently occurring branch points among all MP trees [35]. 

Maximum parsimony is known for its straightforward mathematical approach and absence of a specific model. It is well suited for data types where designing appropriate evolutionary models is challenging, such as rare features based on genomic rearrangements or unique morphological traits. However, when applied to large datasets, it frequently generates numerous potential rooted trees, rendering comprehensive comparisons unfeasible [28].

### 2.3. Maximum Likelihood (ML) Method

**Figure 5:**
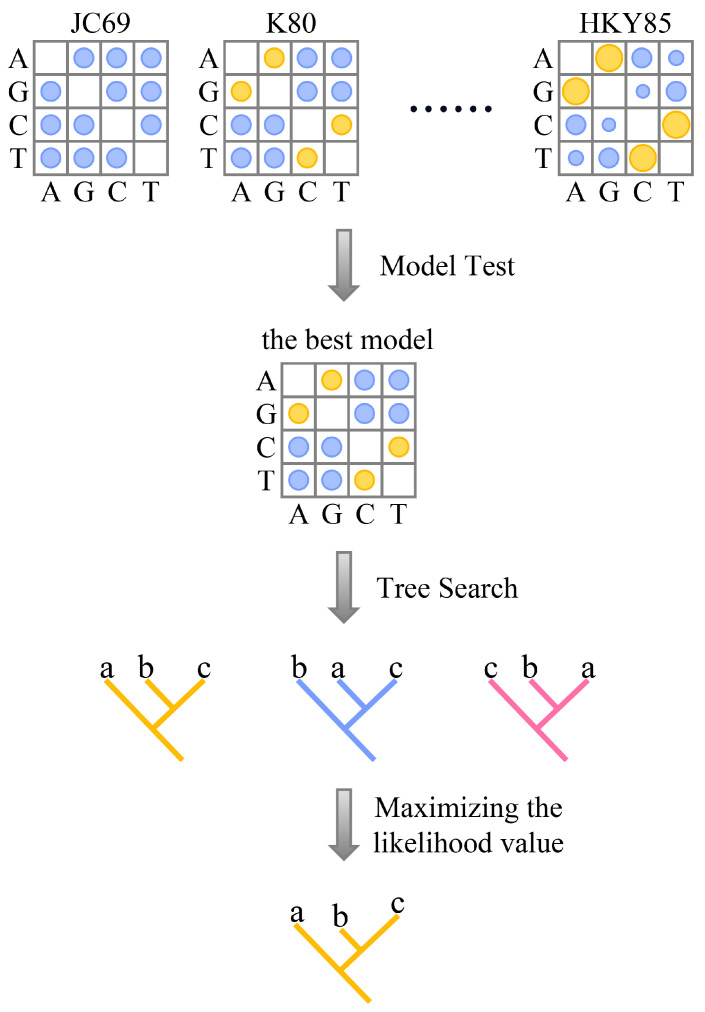
Fundamentals of maximum likelihood method for constructing phylogenetic trees. The abbreviations in the figure are as follows: JC69, the Jukes and Cantor 1969 model; K80, the Kimura 1980 model; HKY85, the Hasegawa, Kishino, and Yano 1985 model; a, b, and c represent different species.

Maximum likelihood (ML) was first proposed by Felsenstein in the early 1980s [41]. The main process of constructing an evolutionary tree using this method is shown in Figure 5.

First, a suitable evolutionary model is selected based on the characteristics of the sequence data being studied. JC69 [42,43], K80 [44], TN93 [45], HKY85 [46], and GTR [47] are commonly used evolutionary models for analyzing DNA sequences. The JC69 model assumes that all nucleotide substitutions occur with equal probability [42,43]. In contrast, the TN93 model assumes that transitions and transversions occur at different rates, and base frequencies are estimated from the data [48]. The GTR model assumes that all nucleotides occur at different frequencies and convert at different rates. Next, a tree space search is conducted, and optimal substitution parameters and branch lengths for each topology are optimized based on standard numerical optimization principles to maximize the likelihood value for each topology [49,50]. Finally, the topology with the highest ML value is selected as the optimal evolutionary tree. In principle, this step must be repeated for all possible trees to find the maximum likelihood value, but the number of hypothetical trees with n taxa increases rapidly with n. This means that exhaustive searches are only suitable for phylogenetic inference based on a small number of taxa, and for inference based on more taxa, tree space searches are usually heuristic [49]. 

Because likelihood methods have clear model assumptions, the probability of systematic errors (such as long-branch attraction artifacts) is lower than that of parsimony methods. However, the complex model settings greatly increase the computational burden. Maximum likelihood is a statistical method based on evolutionary models. It has advantages such as statistical consistency, robustness, and the ability to compare different trees and make full use of original data within a statistical framework. 

### 2.4. Bayesian Inference (BI) Method

**Figure 6:**
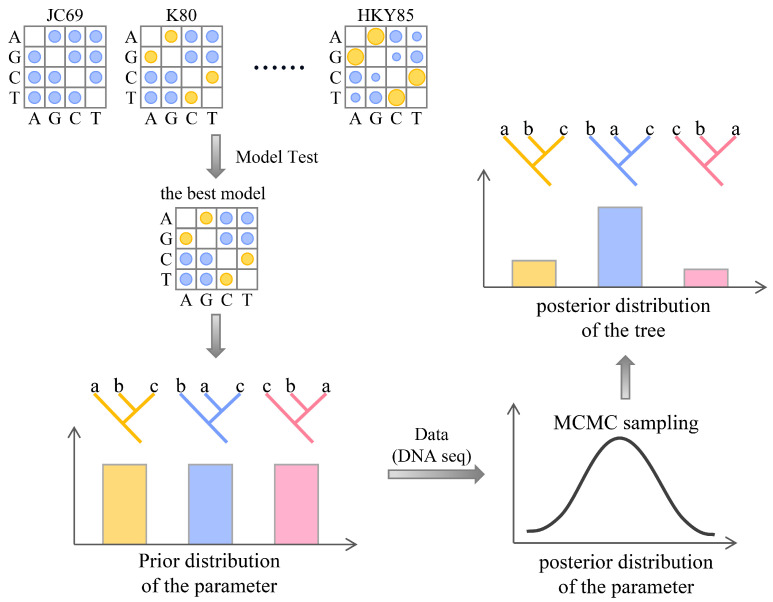
The principles of the Bayesian inference method for constructing phylogenetic trees. The abbreviations in the figure are as follows: JC69, the Jukes and Cantor 1969 model; K80, the Kimura 1980 model; HKY85, the Hasegawa, Kishino, and Yano 1985 model; MCMC, Markov chain Monte Carlo; a, b, and c represent different species.

Bayesian inference (BI) for phylogenetic inference was proposed by Bruce Rannala and Ziheng Yang in the 1990s [51,52]. Its appearance changed the way people analyze genomic sequences [53]. Unlike ML methods, Bayesian methods use statistical distributions to quantify uncertainty in parameters [28]. The main process of tree construction is shown in Figure 6. First, a suitable evolutionary model is selected for the sequence being studied, and parameter prior information (such as tree topology and branch length) is reasonably set based on professional knowledge and experience [54]. Most phylogenetic models use continuous-time Markov processes (CTMPs) to model nucleotide substitution, which have an important property called the Markov property: the future state (remaining time before the next substitution and the character state produced by the next substitution) depends only on the current state and is independent of the past states [49]. 

According to Bayes’ theorem, combining the prior information of parameters with the likelihood of sequence data can obtain posterior information of parameters, i.e., the posterior probability distribution of parameters. Then, MCMC sampling is conducted: random samples of parameters are obtained from the posterior probability distribution, and a phylogenetic tree is constructed based on each sample. This set of samples forms a Markov chain, which converges to a stationary distribution that is equal to the posterior distribution. The most commonly used MCMC algorithms include the Metropolis–Hastings algorithm [55], Metropolis-coupled MCMC [56], and Larget and Simon’s LOCAL algorithm [57]. The posterior probability distribution of trees can be approximated by the proportion of times each tree is sampled during MCMC sampling. Similarly, the posterior probability of a branch can be estimated by the proportion of sample trees that include that branch [58]. Finally, the topology with the highest posterior probability is selected as the optimal tree.

The superiority of Bayesian inference lies in its ability to handle large datasets at a higher computational speed than maximum likelihood methods and to measure the confidence of trees through posterior probabilities.

## 3. Advanced Computational Integrative Methods for Inferring Phylogenetic Tree

When constructing phylogenetic trees, some researchers construct trees based on individual gene (or protein) sequences, while others combine multiple gene (or protein) sequences to build a phylogenetic tree together [59,60]. For a specific group of species, phylogenetic trees constructed from individual genes often show inconsistency with each other [61,62]. As the number of taxa increases, single-gene phylogenetic trees typically have low statistical support [63]. Studies have reported that when using the same parameters and the same program, approximately 9% to 18% of single-gene phylogenetic trees cannot replicate the same topology [64].

**Figure 7:**
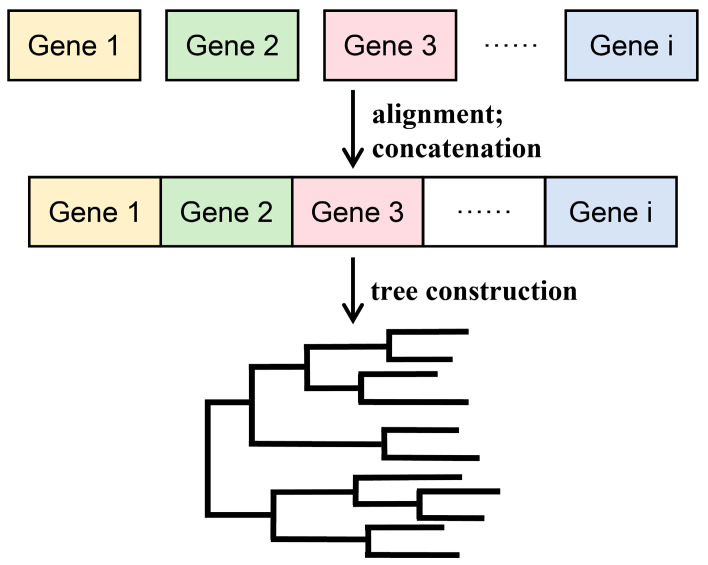
Strategies and principles of the concatenation phylogeny method for constructing phylogenetic trees.

**Figure 8:**
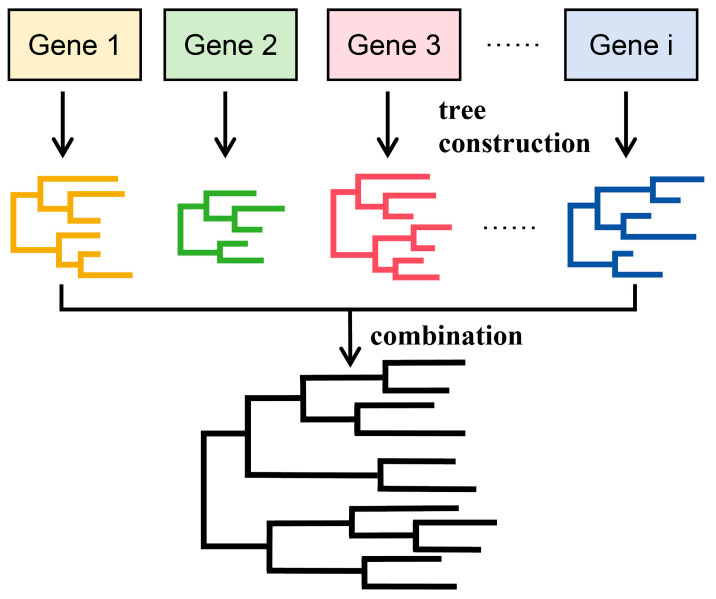
Strategies and principles of the coalescence phylogeny method for constructing phylogenetic trees.

Different genes have different evolutionary rates and evolutionary times and contain varying amounts of informative sites with different resolutions. Combining multiple gene fragments (loci) for phylogenetic analysis can provide more accurate information and higher tree resolution compared to analyzing a single gene [65,66], so the combination of multiple gene sequences has become the mainstream approach in phylogenetic studies [67]. Currently, there are two main methods for constructing multi-gene phylogenetic trees: concatenation phylogeny and coalescence phylogeny [28,68]. The prerequisite for implementing these methods is sequence alignment, and the main processes are shown in Figure 7 and Figure 8, respectively.

### 3.1. Concatenation Phylogeny Method

As shown in Figure 7, concatenation phylogeny, also known as the supermatrix method or total evidence, is a method of constructing a phylogenetic tree by concatenating different gene sequences that have been aligned into a supergene matrix [69]. When following the principle of using all available data (total evidence), the most popular strategy is to use the standard molecular sequence-based methods to analyze single-gene concatenations into a “super gene” [70]. Combining multiple gene fragments for phylogenetic analysis can provide more accurate information than analyzing a single gene and can reveal hidden phylogenetic information in the data [71]. The supermatrix method fully utilizes all information from each dataset and is stable against missing data [72], but implementing this method requires all genes to have the same set of taxa. In addition, the supermatrix method usually assumes that all genes have undergone the same evolutionary process, while there may be lineage sorting during the evolution of species, which can lead to conflicts between gene trees and species trees [71]. 

### 3.2. Coalescence Phylogeny Method

As shown in Figure 8, the coalescence phylogeny method, also known as the supertree method or separate analysis, first independently analyzes each aligned gene to provide estimates for single gene trees. These individual subset trees are then integrated into a single phylogenetic tree to represent the final phylogenetic analysis. The integrated evolutionary tree will include all taxa from the source data set. Commonly used methods for integrating trees include the matrix representation with parsimony (MRP) [73,74], strict consensus [75,76], semi-strict consensus [77,78], and average consensus procedure [79,80], among which MRP is the most popular. 

Unlike the supermatrix method, the supertree method only requires a partial overlap of taxa between different data sets. In cases of incomplete sampling, the results obtained from the supertree method are usually superior to those from the supermatrix method [81]. Studies across multiple data sets have shown that multi-species supertree models generally outperform concatenation models in phylogenetic inference [82]. However, because the supertree method directly operates on the phylogenetic tree and utilizes tree information summarized from various data sets, it often overlooks a significant amount of phylogenetic information [71].

## 4. Construction and Evaluation of Phylogenetic Trees in R Language Environment

The construction of phylogenetic trees can be achieved using various methods such as local software, online tools, and programmable code. Currently popular tree-building software includes PHYLIP [83], PAUP* [84], PhyML [85], MrBayes [86], MEGA [87], and Phylosuite [88]. These software packages typically include multiple algorithms, models, and related analysis functions (such as model comparison and bootstrap analysis), making it easier for users to perform different types of phylogenetic tree reconstruction and evolutionary analysis. However, due to the preset nature of software functionalities and options, it is often challenging to meet users’ flexible analysis needs. Processing large datasets with these software tools can be cumbersome and slow, leading to various inconveniences.

In contrast, scientific programming languages like R and Python provide rich scientific computing and data analysis libraries. R is an open-source software used for statistical analysis and graphic plotting [89,90], making it particularly suitable for phylogenetic tree construction, visualization, and in-depth analysis [91]. With an extensive and vibrant user community, R fosters a collaborative environment for exchanging support, sharing experiences, and solving problems among its members. R offers a wide range of packages tailored for phylogenetic tree construction and analysis, including popular packages such as ape [92], phangorn [93], and dendextend [94], giving users greater convenience and flexibility. There is also a wealth of R packages dedicated to algorithm selection, method exploration, robust data processing, and visualization capabilities. Notable examples include Treeio [95] and tidytree [96], which facilitate the manipulation of evolutionary trees and associated data within R. In addition, ggtree [97] and ggplot2 [98] serve to enhance the visual aesthetics of phylogenetic trees while maintaining their interpretive clarity. Using the R environment for phylogenetic tree construction allows users to customize parameters to suit specific research needs. 

In addition to R, Python stands out as a powerful tool for performing phylogenetic analysis, with an extensive set of libraries and tools such as Biopython [99] and DendroPy [100] that provide a wide range of functions and algorithms. In addition, Python provides access to machine learning and deep learning libraries such as scikit-learn [101] and PyTorch [102], which facilitate effective model building and prediction in phylogenetic analysis. By programming automated reconstruction of phylogenetic trees and batch processing of large datasets, researchers can significantly improve the efficiency of their analyses. It should be noted that this approach can involve more coding and debugging and requires a certain level of programming skills, which can be a learning curve for non-experts. 

**Figure 9:**
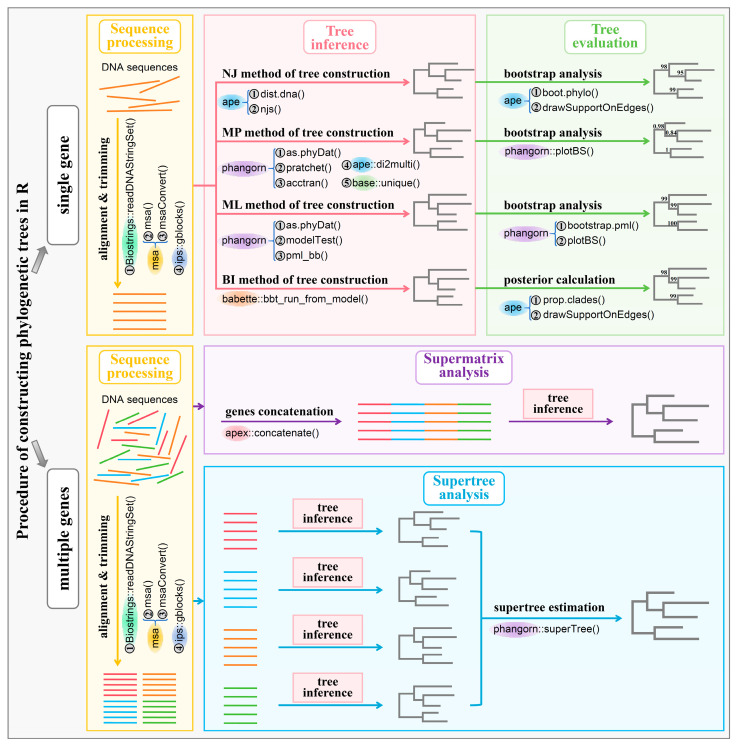
The whole procedure for building the phylogenetic trees in R environment. The abbreviations in the figure are as follows: NJ, neighbor-joining; MP, maximum parsimony; ML, maximum likelihood; BI, Bayesian inference.

Figure 9 shows the whole procedure for building the phylogenetic trees in the R environment. In this review, we have selected sixteen model species, including *Homo sapiens* (human), *Mus musculus* (mouse), *Rattus norvegicus* (rat), *Drosophila melanogaster* (fruit fly), *Arabidopsis thaliana* (thale cress), *Saccharomyces cerevisiae* (brewer’s yeast), *Macaca mulatta* (rhesus monkey), *Caenorhabditis elegans* (roundworm), *Sus scrofa* (pig), *Bos taurus* (cow), *Gallus gallus* (chicken), *Zea mays* (corn), *Oryza sativa* (rice), *Escherichia coli* (*E. coli*), *Glycine max* (soybean), and *Xenopus laevis* (African clawed frog). We will download their orthologous gene K00927 sequences and use them as examples to construct phylogenetic trees using the NJ method, MP method, ML method, and BI method.

Firstly, the DNA sequence files of the orthologous gene K00927 for the mentioned 16 model organisms were downloaded from the gene database under NCBI. After sequence alignment, we use the *fasta2DNAbin()* function in the R package adegenet [103] to read alignments with the FASTA format and convert them into DNAbin objects. 

### 4.1. Implementation of Distance-Based Methods in R

The NJ method can be implemented using relevant functions in the ape package. The ape package is primarily used for reading, writing, analyzing, and simulating phylogenetic trees and DNA sequences, calculating DNA distances, translating DNA sequences into protein sequences, and estimating phylogenetic trees using distance-based methods for evaluation [92]. Listing 1 exhibits the complete process of building a phylogenetic tree using the NJ method under the R programming environment. After installing and loading the ape package, the *dist.dna()* function is used to generate the distance matrix of DNA sequences. Users can select the desired molecular evolution model by setting the model parameter, with the default being the K80 model. Based on this distance matrix (which can contain missing values), the phylogenetic tree (unrooted tree) is constructed using the *njs()* function. The *root()* function is used to set *Escherichia coli* (ece) as an outgroup to define the root. To convert the phylogenetic tree properties to a rooted tree, the parameter r = TRUE needs to be set. Subsequently, *boot.phylo()* function is used for bootstrap analysis, visualized using the *plot()* function, and bootstrap values are added using the *drawSupportOnEdges()* function.

**Listing** **1.** The code for implementation of the neighbor-joining method in R.

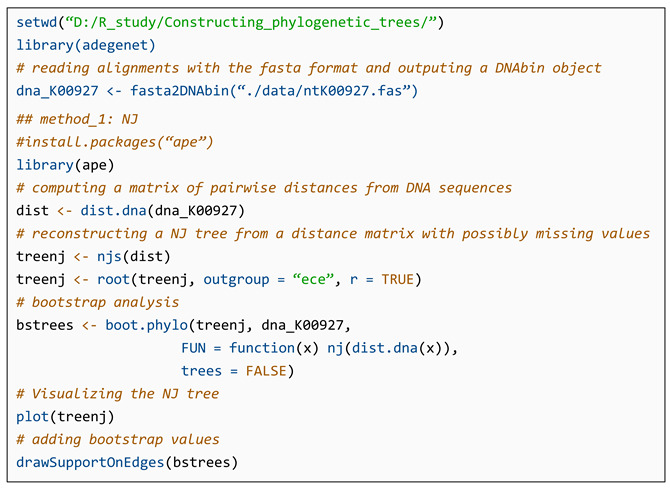

### 4.2. Implementation of MP Method in R

The R package “phangorn” allows for the estimation of phylogenetic trees and networks using maximum likelihood, maximum parsimony, distance-based, and Hadamard conjugation methods. It also provides methods for tree comparison, model selection, and visualization of phylogenetic networks [93]. Listing 2 shows the workflow of building a phylogenetic tree using the MP method under the R environment.

**Listing** **2.** The code for implementation of the maximum parsimony method in R.

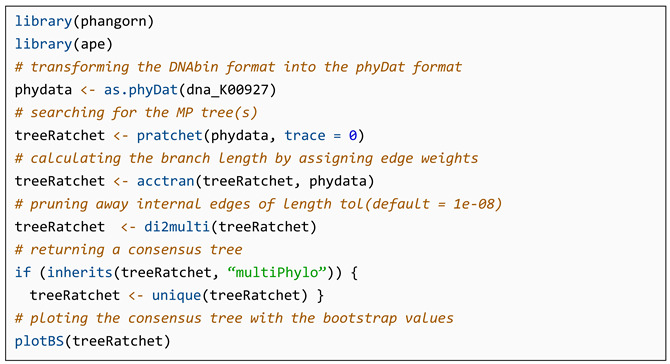

After installing and calling the phangorn package, DNA sequences are converted from DNAbin format to phydata format using the *as.phyDat()* function. In this study, the parsimony ratchet method is applied to search for maximum parsimony (MP) trees using the *pratchet()* function. The minit parameter can be set to determine the minimum number of iterations, and trace = 0 is used to prevent the current state from being written to the console, which does not affect the results obtained from this function. Subsequently, branch lengths are calculated using the *acctran()* function. As the parsimony ratchet method may produce multiple MP trees, the *unique()* function can be used to generate a consensus tree. Finally, the *plotBS()* function is used to visualize the consensus tree and add bootstrap values. 

### 4.3. Implementation of ML Method in R

The maximum likelihood method will also be implemented using relevant functions in the phangorn package. Listing 3 shows the workflow of building a phylogenetic tree using the ML method under the R environment. After converting DNA sequences from DNAbin format to phydata format, the *modelTest()* function is used to perform model testing in search of the best model. Users can compare evolutionary models by setting the model parameter. Subsequently, the *pml_bb()* function is used to infer the maximum likelihood tree using the ML method. Finally, bootstrap analysis is performed, and the tree topology is separated from the output results for visualization.

**Listing** **3.** The code for implementation of the maximum likelihood method in R.

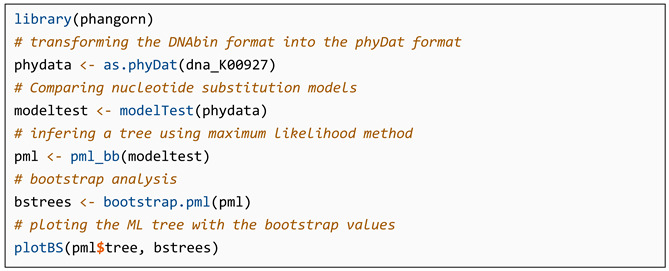

### 4.4. Implementation of BI Method in R

The R package “babette” is a popular alternative workflow for the Bayesian inference software BEAST2. It generates posterior distributions for phylogenetic trees and parameter estimates based on alignment results and inference models [104]. Listing 4 shows the workflow of building a phylogenetic tree using the BI method under the R environment. After installing and calling the babette package, Bayesian inference can be directly performed using the *bbt_run_from_model()* function. The length of the Markov chain Monte Carlo (MCMC), the number of iterations per tree sample (minimum 1000), and other settings can be adjusted using the MCMC parameter in the *create_inference_model()* function. Subsequently, the hypothesis sample with the highest posterior probability is extracted as the optimal tree, and posterior probabilities for branches are calculated, followed by visualization.

**Listing** **4.** The code for implementation of the Bayesian inference method in R.

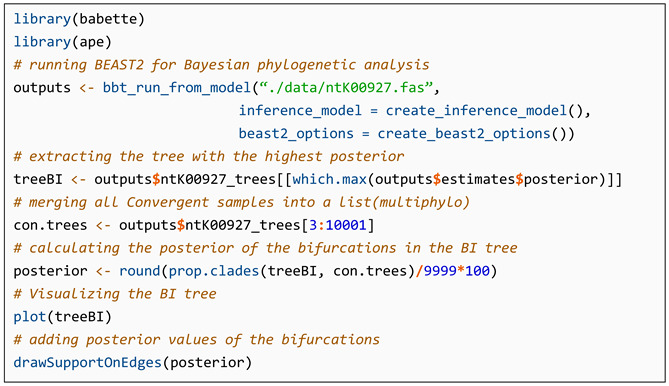

### 4.5. Building the Consensus Phylogenetic Tree Using Multiple Genes in R

For the aforementioned sixteen species we downloaded 10 sets of orthologous genes (K01939, K03644, K00797, K00826, K00088, K02257, K00164, K00820, K06158, and K00008), and used them as input data to construct phylogenetic trees via supermatrix and supertree methods, respectively.

To implement the supermatrix (i.e., concatenation phylogeny method) in the R environment (as shown in Listing 5), the apex package and its dependent devtools package need to be installed first. After aligning the sequences, the FASTA format of these 10 orthologous genes is converted to DNAbin format, merged into a list, and then transformed into multidna format. Subsequently, they are concatenated into a “super gene” matrix, and a phylogenetic tree is constructed using the maximum likelihood method. The core of the supermatrix method lies in concatenating different gene sequences end-to-end to form a complete sequence, which can be understood as combining the sequences of ten genes into a single gene.

**Listing** **5.** The code for implementation of concatenation phylogeny method in R.

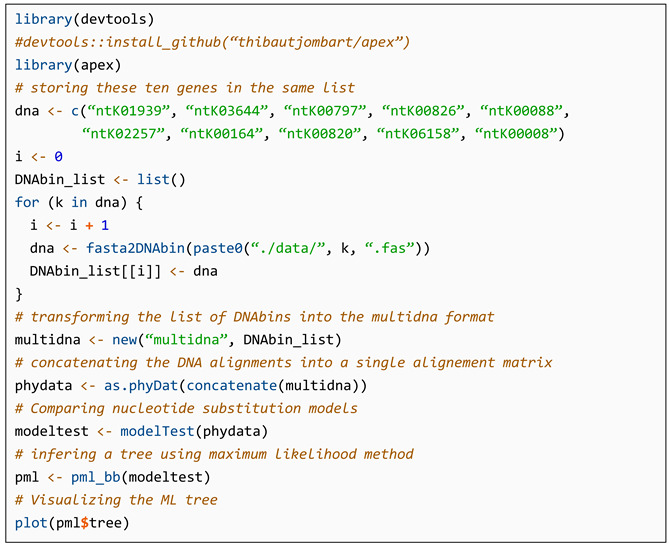

The supertree (i.e., coalescence phylogeny method) is implemented using the phangorn package, with the *superTree()* function being the core function for constructing the supertree. After constructing all individual gene trees based on the maximum likelihood method, they are merged into a multiPhylo format. Then the *superTree()* function is used to integrate them into a single supertree using the MRP method. The whole procedure was illustrated in Listing 6. The core of the supertree method lies in integrating different individual gene trees into a single phylogenetic tree.

**Listing** **6.** The code for implementation of coalescence phylogeny method in R.

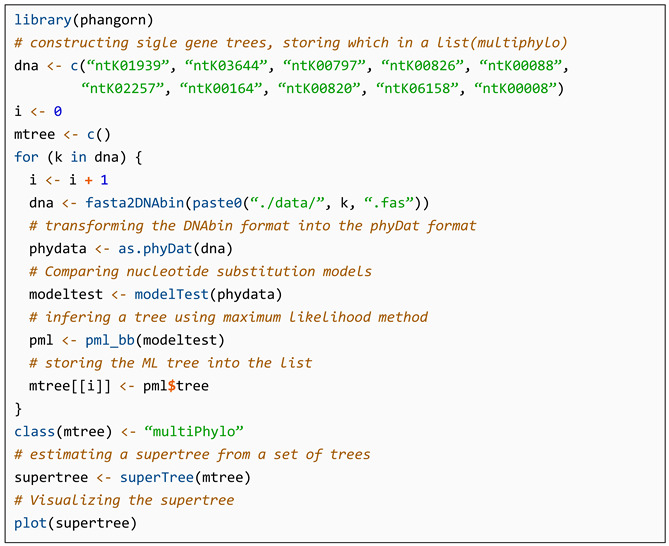

## 5. Summary and Perspectives

Developing methods for constructing phylogenetic trees for different purposes (such as theoretical method innovation and faster computing speed) is an important research field. Phylogenetic trees can intuitively reflect the evolutionary history and relationships among taxonomic units, helping us understand biodiversity and evolutionary patterns. R language is a very powerful statistical analysis and plotting tool, providing many packages for constructing and analyzing phylogenetic trees. Table 2 lists the common R packages used in phylogenetic tree construction. This article reviews the methods for constructing phylogenetic trees and their implementation in the R environment. Through discussions of distance methods, maximum parsimony, maximum likelihood, Bayesian inference, and phylogenetic tree integration methods, and comprehensive analysis of the advantages, disadvantages, and applicable scenarios of different methods, this article provides a reference basis for researchers to choose appropriate methods. Meanwhile, by demonstrating how to use R packages and algorithms to construct phylogenetic trees and providing code examples and practical cases, readers can better understand and apply the knowledge learned.

It is worth noting that with the rapid increase in high-throughput data such as genomics and proteomics, the methods for constructing phylogenetic trees also need to be constantly updated and improved. Currently, new methods are constantly emerging, providing research directions for phylogenetic analysis. Fusang, proposed by Wang et al. [22], is a framework for reconstructing the topology of phylogenetic trees (without calculating branch lengths) through deep learning methods, aiming to provide an evolvable toolkit for daily phylogenetic tree inference applications. However, when there are too many species (>40), the computational efficiency of the current version of Fusang is low, and it currently only supports amino acid multisequence alignments with fewer than 40 sequences and sequence lengths below 10,000. Fusang uses an improved stepwise addition algorithm inspired by Zou et al. [105] to solve variable MSA sequences and uses beam search to find the best topology based on the probability distribution provided by deep learning. When there are more insertions and deletions in the multiple sequence alignment, DL performs better than ML methods because DL adds indel information to greatly improve the accuracy of phylogenetic inference. The authors have also pushed related Python code on GitHub for users to use Fusang.

**Table 2:** The common R packages used in phylogenetic tree construction.

R Package	Description	Source	Reference
ape	Providing both utility functions for reading and writing data and manipulating phylogenetic trees, as well as several advanced methods for phylogenetic and evolutionary analysis.	CRAN *	[92]
phangorn	Estimating phylogenetic trees and networks using maximum likelihood, maximum parsimony, distance methods, and Hadamard conjugation; offering methods for tree comparison, model selection, and visualization of phylogenetic networks.	CRAN *	[93]
babette	Providing an alternative workflow to the BEAST2; conducting complex Bayesian phylogenetics easily and reproducibly from R.	Github	[106]
BAMMtools	Reconstructing and visualizing changes in evolutionary rates through time and across clades in a Bayesian statistical framework.	CRAN *	[107]
apex	Implementing new object classes for storing and handling multiple genes data.	CRAN *	[108]
phytools	Concentrating on phylogenetic comparative biology; including numerous techniques for visualizing, analyzing, manipulating, reading or writing, and inferring phylogenetic trees.	CRAN *	[109]
ggtree	Annotating phylogenetic trees with their associated data of different types and from various sources.	Bioconductor	[97]
RPANDA	Characterizing and comparing phylogenies using spectral densities; fitting models of diversification to phylogenies.	CRAN *	[110]
TreeSearch	Dataset construction and validation; phylogenetic search (including with inapplicable data); the interrogation of optimal tree sets.	CRAN *	[111]
paleotree	Analyzing the combined paleontological and phylogenetic data sets, particularly the time-scaling of phylogenetic trees, which include extinct fossil lineages.	CRAN *	[112]
treeman	Containing a new class called TreeMan for representing phylogenetic trees that has a list structure that allows for more efficient manipulation of phylogenetic trees; demonstrating intuitive tree manipulation, both conceptually and as computationally efficient as possible, within the R environment.	Github	[113]

* CRAN: The Comprehensive R Archive Network.

ModelRevelator, proposed by Burgstaller-Muehlbacher et al. [114], is a machine learning method supported by two neural networks (deep learning) for model selection in phylogenetic inference, which aims to find the best model for sequence evolution and tends to choose models with fewer parameters. The authors demonstrated that neural networks can be used for model selection without rebuilding trees, optimizing parameters, or calculating likelihoods. The first neural network, NNmodelfind, recommends one of six commonly used sequence evolution models (JC, K2P, F81, HKY, TN93, and GTR). The second neural network, NNalphafind, provides an estimate of the shape parameter α by suggesting whether to merge gamma-distributed rate heterogeneity models. The authors found that using neural networks for phylogenetic estimation slightly improved compared to ML + BIC and significantly saved computing time depending on the size of the alignment. Users can use ModelRevelator for phylogenetic analysis through IQ-tree software.

Hyperbolic embedding in phylogenetic analysis embeds the sequences of taxonomic groups into hyperbolic spaces using hyperbolic geometry models (the hyperboloid model, Klein disk model, and Poincaré disk model), represented as points, and calculates the distances between them. Hyperbolic space has negative curvature (negative curvature: the sum of interior angles of any triangle on the surface is less than π), and its exponential expansion rate is much greater than that of Euclidean space. Therefore, compared with Euclidean embedding, hyperbolic embedding more closely matches the geometric shape of trees [115] and better represents hierarchical structures [116]. However, hyperbolic embedding is currently significantly more effective than Euclidean embedding only in low dimensions and loses its advantage in high dimensions [117]. Macaulay et al. [118] applied hyperbolic embedding to Bayesian phylogenetic analysis, studying the impact of the curvature (the degree of geometric curvature) and dimension of hyperbolic space on MCMC chain performance, and concluded that hyperbolic embedding allows tree search algorithms to propose new states (topology and branch length) from continuous probability distributions.

New methods of phylogenetic analysis offer distinct advantages over traditional methods but also present challenges that require further refinement by researchers. Future investigations should prioritize gaining a comprehensive understanding of current methods and techniques while critically assessing their limitations, thus providing an essential context for the integration of innovative approaches. Meeting the growing demand for large data sets requires the development of more efficient and accurate methods, as well as deeper investigations into the integration of artificial intelligence and machine learning technologies into phylogenetic tree construction. Encouragingly, the use of advanced large language models (LLMs) [119], such as OpenAI’s ChatGPT [120], known for its exceptional language processing and programming capabilities, offers promising prospects for advancing phylogenetic research. Continued optimization of existing phylogenetic analysis methods and exploration of new techniques within the R programming environment will enable researchers to harness large amounts of data for iterative analysis, resulting in the construction of more robust and comprehensive phylogenetic trees that accurately reflect the evolutionary relationships between species. In addition, this approach may facilitate the identification of minimal orthologous gene sets with whole genome representation.

## Figures and Tables

**Table 1:** The common algorithms used in phylogenetic tree construction.

Algorithm	Principle	Hypothesis	Criteria for Selecting the Final Tree	Scope of Application
NJ *	Minimal evolution: Minimizing the total branch length of the phylogenetic tree.	BME branch length estimation model: Ensuring general statistical consistency of minimum length phylogeny and non-negativity of its branch lengths [21].	In the end, only one tree was constructed.	Short sequences with small evolutionary distance and few informative sites.
MP	Maximum-parsimony criterion: Minimize the number of evolutionary steps required to explain the data set.	No model required.	The phylogenetic tree with the smallest number of base (or amino acid) substitutions during evolution.	Sequences with high sequence similarity, sequences for which it is difficult to design appropriate characteristic evolution models.
ML	Maximize likelihood value.	The sites in the alignment are independent; each branch is allowed to evolve at different rates.	Phylogenetic tree with maximum likelihood value.	Distantly related and small number of sequences.
BI	Bayes theorem.	Continuous-time Markov substitution model: Substitution probability is only related to the current nucleotide and has nothing to do with past nucleotides.	The most sampled phylogenetic tree in MCMC.	A small number of sequences.

* NJ: a representative method and one of the most popular distance-based methods. The abbreviations in the table are as follows: NJ, neighbor-joining; MP, maximum parsimony; ML, maximum likelihood; BI, Bayesian inference; MCMC, Markov chain Monte Carlo.

## Data Availability

All R codes and datasets used in this review are freely available at https://github.com/libcell/phylosinR (accessed on 4 April 2024).
